# Commercial determinants of health: advertising of alcohol and unhealthy foods during sporting events

**DOI:** 10.2471/BLT.18.220087

**Published:** 2019-02-25

**Authors:** Robin Ireland, Christopher Bunn, Gerda Reith, Matthew Philpott, Simon Capewell, Emma Boyland, Stephanie Chambers

**Affiliations:** a College of Social Sciences,School of Social and Political Sciences, University of Glasgow, 25–29 Bute Gardens, Glasgow G12 8RS Scotland.; b School of Social and Political Sciences, University of Glasgow, Glasgow, Scotland.; c European Healthy Stadia Network, Liverpool, England.; d Department of Public Health and Policy, University of Liverpool, Liverpool, England.; e Department of Psychological Sciences, University of Liverpool, Liverpool, England.; f MRC/CSOSocial and Public Health Sciences Unit, University of Glasgow, Glasgow, Scotland.

## Abstract

Tobacco, alcohol and foods that are high in fat, salt and sugar generate much of the global burden of noncommunicable diseases. We therefore need a better understanding of how these products are promoted.The promotion of tobacco products through sporting events has largely disappeared over the last two decades, but advertising and sponsorship continues bycompanies selling alcohol, unhealthy food and sugar-sweetened beverage. The sponsorship of sporting events such as the Olympic Games, the men’s FIFA World Cup and the men’s European Football Championships in 2016, has received some attention in recent years in the public health literature. Meanwhile, British football and the English Premier League have become global events with which transnational companies are keen to be associated, to promote their brands to international markets. Despite its reach, the English Premier League marketing and sponsorship portfolio has received very little scrutiny from public health advocates. We call for policy-makers and the public health community to formulate an approach to the sponsorship of sporting events, one that accounts for public health concerns.

## Résumé

Le tabac, l'alcool et les aliments riches en graisse, en sel et en sucre génèrent la plus large partie de la charge mondiale des maladies non transmissibles. Il est donc nécessaire de mieux comprendre la manière dont ces produits sont promus. La promotion des produits du tabac dans le cadre d’événements sportifs a largement disparu au cours des vingt dernières années, mais la publicité et le sponsoring par des entreprises qui vendent de l'alcool, des produits alimentaires peu sains et des boissons sucrées sont encore d'actualité. Depuis quelques années, la littérature sur la santé publique commence à porter son attention sur le sponsoring d'événements sportifs, tels que les Jeux olympiques, la Coupe du monde masculine de la FIFA ou encore le Championnat d'Europe de football masculin de 2016. Mais dans le même temps, le football britannique et la *Premier League* anglaise sont devenus des événements mondiaux auxquels les multinationales aiment être associées pour promouvoir leurs marques auprès de marchés internationaux. Malgré leur portée, le marketing et le sponsoring de la *Premier League* anglaise semblent négligés par les défenseurs de la santé publique. Nous appelons les décideurs politiques et la communauté de santé publique à élaborer une approche pour le sponsoring d’événements sportifs qui réponde aux enjeux de santé publique.

## Resumen

El tabaco, el alcohol y los alimentos que son ricos en grasa, como la sal y el azúcar, generan gran parte de la carga mundial de enfermedades no contagiosas. Por tanto, necesitamos una mejor comprensión de la forma en que se promueven estos productos. La promoción de los productos del tabaco a través de eventos deportivos ha desaparecido en gran medida en las últimas dos décadas, pero la publicidad y el patrocinio continúan por parte de las empresas que venden alcohol, alimentos poco saludables y bebidas azucaradas. El patrocinio de eventos deportivos como los Juegos Olímpicos, la Copa Mundial de la FIFA masculina y el Campeonato de Fútbol Europeo del 2016 ha recibido cierta atención en los últimos años en la bibliografía sobre salud pública. Mientras tanto, el fútbol británico y la Premier League inglesa se han convertido en eventos globales con los que las empresas transnacionales están dispuestas a asociarse, para promocionar sus marcas ante los mercados internacionales. A pesar de su alcance, la cartera de comercialización y patrocinio de la Premier League inglesa apenas han sido objeto de escrutinio por parte de los defensores de la salud pública. Pedimos a los responsables de la formulación de políticas y a la comunidad de la salud pública que formulen un enfoque para el patrocinio de eventos deportivos, que tenga en cuenta estas preocupaciones sobre la salud pública.

## ملخص

يسبب كل من التبغ والكحول والأغذية الغنية بالدهون والملح والسكر، الكثير من العبء العالمي الخاص بالأمراض غير المعدية.وعلى ذلك، فإننابحاجة لفهم كيفية ترويج هذه المنتجات.نجد أن الترويجلمنتجات التبغ خلال الأحداث الرياضية قد اختفى إلى حد بعيد على مدى العقدين الماضيين، إلا أن حملات الإعلان والرعاية مستمرة من جانب الشركات التي تبيع الكحوليات، والطعام غير الصحي، والمشروبات المحلاة بالسكر.أولت حملات رعاية الأحداث الرياضية مثل الألعاب الأولمبية، وبطولة كأس العالم لكرة القدم للرجال، وبطولة الأمم الأوروبية للرجال في عام 2016، بعض الاهتمام في السنوات الأخيرة للأمور المتعلقة بالصحة العامة.وفي نفس الوقت، أصبحت كرة القدم البريطانية والدوري الإنجليزي الممتاز أحداثعالمية تحرص الشركات متعددة الجنسيات على الارتباط بها للترويج لعلامتها التجارية في الأسواقحول العالم.وعلى الرغم من شعبية الدوري الإنجليزي الممتاز، إلا أنحافظة التسويق والرعاية لم تحصلإلا على القليل من الفحص من جانب المهتمين بالصحة العامة.من هذا المنطلق، نحن ندعو واضعي السياسات ومجتمع الصحة العامة لوضعأسلوب لرعاية الأحداث الرياضية، بحيث يبرز هذا الأسلوب الأولويات الصحية العامة.

## 摘要

烟草、酒类和高脂、高盐、高糖食物会导致全球非传染性疾病负担加重。因此，我们有必要更好地了解这些产品的促销方式。尽管在过去二十多年里，通过体育赛事推广烟草产品的行为已经在很大程度上消失，但酒类、不健康食物和含糖饮料行业的广告宣传和赞助活动却依然存在，这让人们更加担心其对公共健康产生的潜在影响。奥运会、国际足联男子世界杯和 2016 年男子欧洲足球锦标赛等体育赛事的赞助活动在近年来的公共卫生文献中备受关注。与此同时，跨国公司热衷于将自己的品牌通过英国足球和英超联赛这类全球性赛事推向全球市场。考虑到英超联赛触及的范围，其市场营销和赞助系列产品却很少受到公共卫生倡导者认真彻底的审查。我们呼吁政策制定者和公共卫生社会团体制定出一种将公共健康问题纳入考量的体育赛事赞助方法。

## Резюме

Табак, алкоголь и пищевые продукты с высоким содержанием жиров, соли и сахара служат причиной большей части мирового бремени неинфекционных заболеваний. Следовательно, нам нужно хорошо понимать, как рекламируются такие продукты. Продвижение табачных изделий через спорт значительно сократилось за последние два десятилетия, но рекламное сопровождение и поддержка спорта производителями алкоголя, нездоровой пищи и сахаросодержащих безалкогольных напитков продолжается. В последнее время в литературе, посвященной здоровью общества, затрагивались вопросы спонсорской поддержки таких спортивных событий, как Олимпийские игры, чемпионат мира по футболу среди мужчин и чемпионат Европы по футболу среди мужчин в 2016 году. Между тем британский футбол и Английская премьер-лига стали глобальными брендами, с которыми транснациональные компании стремились ассоциироваться для рекламы своей продукции на международном рынке. Несмотря на свою популярность, Английская премьер-лига и ее спонсорское портфолио практически не рассматривались сторонниками защиты общественного здоровья. Авторы призывают ответственных за принятие стратегических решений и сообщество общественного здравоохранения сформировать подход к спонсорству спортивных мероприятий, который бы учитывал проблемы здоровья общества.

## Introduction

Noncommunicable diseases, including cardiovascular and respiratory diseases, cancers and type 2 diabetes, cause an estimated 41 million deaths per year globally, of which 15 million occur between the ages of 30 to 70 years. However, most of these premature deathsare avoidable and noncommunicable disease prevention is thus a global priority. The main prevention strategies focus on the risks associated with poor diet, tobacco use, alcohol consumption and physical inactivity.^1^As evidence on the social determinants of health has become critical to the understanding of noncommunicable disease epidemiology, we also need to consider the commercial determinants of health when developingrisk reduction strategies.^2^ In noncommunicable disease prevention, an emphasis is often placed on lifestyles^3^ and personal responsibility for addressing risk factors. This approach ignores the limited control that many people have over their circumstances and their exposure to the marketing activities of transnational corporations.^4^

Sport is often presented as a way for people to lead more active, healthier lives. Yet many sports have become closely entwined with products that harm health. Companies producing alcohol, sugar-sweetened beverages, and foods high in fat, sugar and salt, often market their products through professional sports leagues, in competitions and events across the world.^5^ We know that consumption of these products contributes to the global burden of noncommunicable disease.^6^ We now need to better understand the role of corporate marketing and sponsorship strategies in their promotion of such products.

We therefore apply a public health perspective to the commercial sponsorship of sport. We suggest that policy-makers who wish to reverse the noncommunicable disease burden should consider how sport has been used to promote productsthat harm health and whether regulation may be required to control this marketing.

## Commercial determinants of health

Health is not only determined by biological and genetic factors, but by the socioeconomic context of people’s lives, including income levels and educational standards.^7^Corporate activity, such as marketing of harmful goods including unhealthy foods, tobacco, sugar-sweetened beverages and alcohol, also affects health.^8^Commercial determinants of health are defined as “factors that influence health which stem from the profit motive.”^9^ Corporate activities shape our environments and determine the availability, promotion and pricing of consumables.^10^

International sporting events like the Olympic Games and the *Fédération Internationale de Football Association* (FIFA) World Cup provide transnational corporations with large platforms to market their products.^11^ For example, despite a Brazilian law adopted in 2003 prohibiting the sale of alcoholic drinks at sporting events, FIFA overruled the law for the 2014 World Cup in Brazil by stating that access to beer was non-negotiable.^12^ Jérôme Valcke, FIFA’s former Secretary General, said that “alcoholic drinks are part of the FIFA World Cup, so we’re going to have them. […] The fact that we have the right to sell beer has to be part of the law.”^13^ Budweiser® was, and remains, a sponsor of the FIFA World Cup, including the 2018 tournament hosted in the Russian Federation.

## Commercial sponsorships

The link between the Olympic Games and corporations can be traced back at least to the 1928 games in Amsterdam, when organizers recognized the commercial potential of the event. Coca-Cola® kiosks were staffed by vendors displaying Coca-Cola® branding.^14^ By the 1970s, the then president of FIFA, was taking advantage of the World Cup’s global television market to develop corporate sponsorship. This sponsorship was segmented by product type, including Coca-Cola® as the recognized sweetened beverage partner from 1978 and with Budweiser® as the official beer sponsor from 1986.^15^

However, public health professionals have rarely noted the ethical issues and conflicts of interest involved in the commercial sponsorship of the games until recent events like the 2012 Olympic and Paralympic Games.^16^ Since then, major sports events have begun to draw scrutiny from a health perspective. Assessing the food and drinks promoted at the 2016 Union of European Football Associations (UEFA) Championship, researchers noted that unhealthy food and drink products dominated inside the stadia and sponsors included companies such as Coca-Cola®, McDonald’s and Carlsberg Group.^17^

## Sport and tobacco

Since the 1960s, the awareness of the harms caused by tobacco has been growing, resulting in increasingly restrictive legislation on direct tobacco advertising. Sport sponsorship provided an opportunity for cigarette companies to achieve brand exposure across a range of sports worldwide while circumventing this legislation.^18^For example, Philip Morris International used the women’s tennis tour to promote their Virginia Slims brand throughout the 1970s and 1980s.^19^ Motor sports were strongly associated with tobacco sponsorship from 1968.^20^The adventurous image of motor sports matched tobacco companies’ objectives and brands that targeted young adult male urban smokers.^18^ The tobacco industry’s sponsorship strategy seemed effective, as a study of a tobacco company’s sponsorship of the India-New Zealand cricket series in 1996 showed the likelihood of Indian children experimenting with tobacco almost doubled as a result of watching the matches on television.^21^ The World Health Organization (WHO) addressed tobacco sponsorship with a“Tobacco Free Sports” campaign in 2002.^22^In 2003, WHO Member States endorsed the WHO’s Framework Convention on Tobacco Control (FCTC).^23^ Article 13 of the FCTC enacts a comprehensive ban on tobacco advertising, promotion and sponsorship including through sport.

Public health practitioners refer to tobacco control as a success story in reducing cigarette consumption and thus directly reducing the prevalence of cardiovascular and respiratory diseases in many countries.^24^Much can be learnt from tobacco control, because the strategies used by the tobacco industry to delay or prevent regulation are now being used by some food and alcohol companies. These strategies include: giving large sums of money to politicians and journalists; attacking public health champions; recruiting commercial allies; misinformation via propaganda and poorly conducted science; and substituting strong interventions with weak ones.^7^^,^^25^For example, the relationship between Coca-Cola® and the Global Energy Balance Network has raised concerns about food and beverage corporations’ involvement with scientific organizations where they may be seen to promote business interests. Emails sent by Coca-Cola® are reported as suggesting they could “change the conversation about obesity” despite the evidence.^26^

## Relationships with industry

Although food and non-alcoholic beverage companies now invest large sums in marketing through professional sports events,^27^ the impactof this strategy on noncommunicable disease risks has not been quantified.^28^ A systematic review of food and beverage marketing to children through sport showed that although there is clear evidence that food marketing influences children’s choices, research on this type of marketing in sport is limited.^5^ The risk of children being exposed to high amounts of unhealthy food advertising on television was acknowledged and partly addressed by a ban in the United Kingdom of Great Britain and Northern Ireland on the advertising of foods high in fat, sugar and salt during broadcasting of children’s television programmes. However, this ban is only implemented on programmes mainly appealing to children aged 4–15 years.^29^ Sports programmes are not included in this categorization. Other countries, such as Australia, France and Norway, have also addressed advertising on children’s television through various regulatory approaches.

Research from Australia shows that children easily identify unhealthy food and alcohol brands from sponsorship of sporting events, and this recognition influences children’s behaviour.^30^Researchers have documented the techniques used by industry to appeal to children, and to ensure that childrenassociate sport with these products from an early age.^30^^,^^31^ In United Kingdom of Great Britain and Northern Ireland, researchers have noted that televised English Premier League football matches carry advertisements foralcohol and unhealthy foods.^32^

Alcohol has long been associated with sport. In France, the national law *LoiÉvin*, passed by the government in 1991, restricted tobacco and alcohol advertising. Despite this law, companies were unwilling to abandon French sporting events as they saw fans as a key demographic.^33^ Research on alcohol marketing during the UEFA Euro 2016 football tournament held in France,^34^ showed that despite the *LoiÉvin,* the tournament sponsor, Carlsberg Group, was able to achieve a substantial number of alcohol marketing references per televised match. Most references were indirect and used the phrases “probably” or “the best in the world.” The colour of the text was white on a green background, which is associated with Carlsberg beer. This so-called alibi marketing has previously been used by the tobacco industry to circumvent advertising regulations in which, although a brand is not directly mentioned, brand association can be achieved through textual or visual referencing. 

When regulation is inconsistent,companies may argue that they are placed at a commercial disadvantage. For example, some people argued that the *LoiÉvin* is putting French football clubs at a disadvantage in comparison to those in neighbouring countries. The researchers noted that as well as sponsoring revenues, annual revenue from beer sales was 40 million euros for the football clubs in the German Bundesliga.^35^^,^^36^

Digital media provide new ways to market and sell products and toevade marketing regulations. For example, a digital overlay technique applied to perimeter advertising boards in sports stadia allow virtual brand messages to be inserted during ongoing live broadcasting and these messages are visible only to the television broadcasting audience in targeted territories.^37^Online platforms, such as Facebook and Twitter, make it possible to include advertising in social media strategies. Some sport clubs have established a broad base of followers.^38^In February 2019, Manchester United Football Club had 18.8 million followers on Twitter and its Facebook page had 73.3 million likes. However, aggressive marketing through these channels may not be acceptable to fans, reducing club revenue.^39^

## Football and the global community

Many sporting events have a global reach. Football is the most popular spectator sport globally. Televised matches of the English Premier Leagueattractapproximately 4.7 billion views per season.^40^ By sponsoring football teams and by advertising at matches and during commercial breaks, companies can achieve huge exposure for their products and brands.

Companies are increasingly expected to demonstrate a greater commitment and contribution to society through social and environmental activities, characterized as corporate social responsibility. The sports industry has also started addressing its wider social responsibilities.^41^Some football clubs have founded charities that work for the benefit of their local communities. However, the work of these charities may conflict with the aims of the clubs’ sponsors.^42^ There are other governance models of corporate social responsibility in Europe, such as *Verein fürLeibesübungen*(VfL)Wolfsburg in the German Bundesliga.^43^

Given concerns about the prevalence of noncommunicable diseases, public health organizations and academics are starting to question and challenge the commercial relationships within the English Premier League. In 2018, Sugar Smart and the European Healthy Stadia Network wrote to football associations requesting that they decline partnership deals with companies producing foods high in fat, sugar and salt as a commitment to protect children’s health.^44^In Australia, a report published by Cancer Council New South Wales, called for action to reduce children’s exposure to unhealthy food and drink, and alcohol marketing through sponsorship of community and professional sport.^45^

## Conclusion

Professional sport represents a profitable global entertainment industry. Multinational corporations use the visibility and widespread appeal of sports to promote their brands and products to mass audiences. Yet, public health professionals rarely discuss the nature of this influence in professional sport and the methods by which global corporations use sporting events, leagues and clubs to sell products harmful to health. We have focused on football as the world’s most popular sport and the English Premier League in particular. However, other sports also have large numbers of spectators, such as the American National Football League and the National Basketball Association. Sponsors for these sports include the beer company Anheuser-Busch InBev, the restaurant chain Pizza Hut^46^ and the whiskey company Jack Daniel’s.^47^ Similarly, cricket in Australia is often used to promote the products of food and alcohol companies.^48^

The success of removing tobacco from sports sponsorship may inform other public health advocacy measures. However, the tobacco industry developed considerable expertise in circumventingsuch tactics may be used by other companies.

There is clear scope for action by policy-makers to reduce the impact of commercial interests, amplified through sport, on population health. WHO’s Independent High-Level Commission on Noncommunicable Diseases encouraged governments to engage constructively with the private sector (except the tobacco industry). In Recommendation 4 (c), the commission proposes governments should work with “the leisure and sports industries to promote physical activity;” while in 4 (d) the commission states “Governments should give priority to restricting the marketing of unhealthy products (those containing excessive amounts of sugars, sodium, saturated fats and trans fats) to children.”The report also suggested that WHO explore the possibility of establishing an international code of conduct on this issue.^49^^,^^50^ These two recommendations could be considered in tandem. While professional sport has the potential to encourage healthier lifestyles, it is regularly used to sell products, which may impact negatively onhealth. Perhaps WHO should consider an equivalent of its successful “Tobacco Free Sports” campaign^22^ and initiate calls for controls of marketing of unhealthy products within sport.

We encourage research of the relationships between sport and its commercial sponsors, notably the companies producing alcohol, sugar-sweetened beverages, and food high in fat, salt and sugar. All these products present substantial challenges to public health. We suggest that the sports industry embrace a socially-responsible approach to commercial sponsorship and advertising, an approach which emphasises the future health of sports’ fans, families and communities.
